# Bone mineral density in women with polycystic ovary syndrome

**DOI:** 10.1007/s40618-014-0175-5

**Published:** 2014-09-23

**Authors:** K. Katulski, S. Slawek, A. Czyzyk, A. Podfigurna-Stopa, K. Paczkowska, N. Ignaszak, N. Podkowa, B. Meczekalski

**Affiliations:** 1 The Department of Gynecological Endocrinology, Poznan University of Medical Sciences, Polna 33, 60-535 Poznan, Poland; 2 Students Scientific Association of the Department of Gynecological Endocrinology, Poznan University of Medical Sciences, Poznan, Poland

**Keywords:** Polycystic ovary syndrome (PCOS), Bone mineral density (BMD), Z-Score’ hyperandrogenism, HOMA–IR

## Abstract

**Purpose:**

PCOS is a complex disorder and various features of this disorder may have great importance for bone metabolism. The aim of the study was to determine the relationship between existing hormonal disorders, and bone mineral density (BMD) in young women with PCOS.

**Methods:**

69 reproductive-aged PCOS women and 30 age-matched healthy controls were enrolled to the study women. In each individual we assessed the body mass index (BMI). We evaluated the serum concentrations of: gonadotropins, prolactin (PRL), estradiol (E2), dehydroepiandrosterone sulfate (DHEAS), testosterone (T), thyroid stimulating hormone (TSH), free thyroxine (fT4). We used the Homeostatic Model Assessment–Insulin Resistance Index (HOMA–IR) to diagnose insulin resistance. Bone mineral density in the lumbar spine was measured by dual-energy X-ray absorptiometry (DXA).

**Results:**

The PCOS women had lower BMD values as compared to the controls (1.057 ± 0.1260 vs. 1.210 ± 0.1805 g/cm^2^, *p* < 0.0002). In the analysis of PCOS patients according to BMI, only in the subgroup of the normal weight PCOS we find significantly lower BMD in comparison to controls (*p* = 0.0049). In patients with PCOS, BMD was positively correlated with insulin concentration and HOMA–IR. In the controls Z-score values were positively correlated with insulin concentration and HOMA–IR.

**Conclusions:**

The deleterious effect of estrogen deficiency on bones in PCOS is not balanced by androgen overproduction. Women with PCOS had significantly lower BMD of the lumbar spine compared to controls. Insulin seems to be one of the most important positive bone growth stimulators.

## Introduction

Polycystic ovary syndrome (PCOS) is one of the most common disorders in reproductive-aged women and is characterized by oligo- or anovulation, clinical or biochemical hyperandrogenism and polycystic ovarian morphology on ultrasound [1–3]. PCOS is commonly associated with obesity, insulin resistance, dyslipidemia and hypertension [1, 2, 4].

PCOS is a complex disorder and various features of this disorder may have an influence on bone metabolism. Peak bone mass is reached between 25 and 30 years of age, and 45–50 % of peak bone mass is formed during the adolescence period [3]. Peak bone mass is the maximal amount of bone tissue at the end of skeletal maturation, which is an important determinant of the (potential) development of osteoporosis. Many factors are supposed to influence bone deposition during growth [3]. Genetic factors are responsible for about 60–80 %, and hormonal and nutritional factors 40–60 % for the development of peak bone mass [5]. Many previous studies have suggested that a relatively high estrogen concentration, higher insulin concentration, hyperandrogenemia and obesity are crucial bone growth stimulating factors in women with PCOS [6–9].

Estrogens play a key role in the development and maintenance of the appropriate bone mass in women, by acting on osteoblasts, as well as on osteoclasts. The influence of androgens on bone mass has not been fully elucidated. Probably, they stimulate bone development during puberty. The main mechanism of androgen action on bones is believed to be linked to the aromatization of androgens to estrogens in the ovaries and extra glandular tissues. Moreover, all bone-forming cells have receptors for both androgens and estrogens with a predominance of androgen receptors on osteoblast cells [10]. Estrogens and androgens production is significantly altered in PCOS, which probably has great importance for bone metabolism in young women.

Ovulation disorders may also affect bone metabolism in young women. In this regard, the role of progesterone seems to be important [11, 12]. Furthermore, the elevated circulating insulin levels often associated with PCOS may influence the osteoblast cells activity through direct stimulation or indirectly by reducing production of the sex hormone binding globulin (SHBG) and insulin-like growth factor binding protein (IGFBP) [6, 13].

The aim of the study was to determine the relationship between existing hormonal disorders, and bone mineral density parameters (BMD and Z-score) in women with PCOS. Previous publications have reported inconsistent results regarding the impact of hyperandrogenism on the attained bone mass in PCOS.

## Materials and methods

Sixty-nine women of reproductive age with PCOS (mean age 23.13 ± 4.43 years, range 17–34 years) were enrolled to the study. Patients were diagnosed according to the Rotterdam criteria, where two out of three conditions were presented: (1) Oligo and/or anovulation (oligomenorhhea-menstrual cycle between 35–90 days, or secondary amenorrhea–the absence of vaginal bleeding for >90 days); (2) Clinical and/or biochemical signs of hyperandrogenism (modified Ferriman–Gallway score (mFG) ≥8 and/or total testosterone (T) >0.6 ng/ml; (3) Polycystic ovaries (PCO), identified by ultrasonography (US) (presence of ≥12 follicles in each ovary, measuring 2–9 mm in diameter and/or increased ovarian volume >10 cm^3^). Patients with clinical or biochemical features of Cushing syndrome, hyperprolactinemia, ovarian or adrenal virilizing tumors, or non-classical congenital adrenal hyperplasia were excluded from the study [1]. Patients who fulfilled the diagnostic criteria for PCOS were further subcategorized into normal weight or overweight/obese using a body mass index (BMI) cutoff of ≥25 kg/m^2^.

The control group consisted of 30 age-matched healthy women (mean age 21.93 ± 1.23 years, range 20–30 years), who had regular menstrual cycles (between 27 and 35 days in length, for at least the past 6 months). Their health condition was evaluated by medical history, physical and pelvic examination and a basal biochemical examination (heamatocrit, complete blood count, liver enzymes and creatinine). None of the patients or control subjects had received oral contraceptives in the 6 months preceding the study, antidiabetics or had been recommended a restrictive diet.

The body height and weight were measured for each patient at baseline. The BMI was calculated as the ratio of weight and the square of the height. Hirsutism was evaluated by using a modified Ferriman-Gallway score. The Homeostatic Model Assessment–Insulin Resistance Index (HOMA–IR) was calculated using the International Formula: fasting Glucose (mmol/l) × fasting Insulin (mU/l)/22.5.

Blood serum samples, which were obtained from the antecubital area, were collected between 6.00 and 9.00 a.m following 10–12 h of fasting, in the follicular phase, on the 10th day of the cycle. Fasting blood glucose (FBG) was evaluated using an enzymatic UV test (hexokinase method). Serum concentrations of: follicle stimulating hormone (FSH), luteinizing hormone (LH), prolactin (PRL), 17β-estradiol (E2), dehydroepiandrosterone sulfate (DHEAS), testosterone (T), thyroid stimulating hormone (TSH), free thyroxine (fT4) and insulin (Ins) were determined using electrochemiluminescence immunoassay (Roche Diagnostics, USA). Concentrations of various serum hormones were measured using a Cobas E601 analyzer (Roche Diagnostics, USA).

Bone mineral density (BMD) values in the lumbar spine (L1–L4) were measured by the dual X-ray absorptiometry (DXA) method using Lunar DTX ver. 3.65 (Lunar DXP 100, Lunar Corp., Madison, USA). The results are expressed as BMD and the Z-SCORE in absolute values (g/cm^2^). The Z-scores reflect the number of standard deviations by which a patient’s value differs from the age- and gender-matched, normal reference Polish population. Quality control procedures were carried out in accordance with the manufacturer’s recommendations to control for possible baseline drift. The coefficient of variation (CV) for repeated measurement by the DXA operator of the lumbar spine was <1.0 %.

Statistical analysis was performed using the software StatSoft 2011 STATISTICA Version 10. The normality of data distribution was verified with the Shapiro–Wilk test. The variables were verified by tests: the parametric Student’s *t* test or nonparametric Mann–Whitney* U* test. Subgroup analysis of PCOS after stratifying by BMI was performed using a one-way ANOVA and Duncan’s test. A *p* value ≤0.05 was considered significant. Interactions between the variables were tested using Pearson’s linear correlation analysis.

The Local Bioethical Committee approved the study protocol and the patients provided written consent.

## Results

The characteristics of both groups: PCOS and control are presented in Table 1. No significant differences in BMI were found between PCOS and controls.

**Table 1:** Clinical characteristic of PCOS and control groups. No significant differences in BMI were found between PCOS and controls

Group	PCOS	Controls
Number	*n* = 69	*n* = 30
Age (years) (mean ± SD)	23.13 ± 4.43	24.53 ± 2.67
BMI (kg/m^2^) (mean ± SD)	23.07 ± 5.9	21.93 ± 1.23
Normal (BMI 18.5–24.99 kg/m^2^)	*n* = 41	*n* = 30
Obese/overweight (BMI ≥25 kg/m^2^)	*n* = 17	*n* = 0
Eumenorrheic	*n* = 7	*n* = 30
Menstrual irregularities (oligo-/amennorhea)	*n* = 62	*n* = 0

*BMI* body mass index

PCOS women had significantly lower mean levels of FSH (*p* = 0.003). No significant differences were revealed in the LH concentrations between patients and controls [12.8 ± 6.2 U/l in PCOS group and 11.06 ± 1.12 U/l in control group (*p* = 0.73)]. Comparing with the women in the control group, the PCOS women had higher mean serum concentrations of PRL (*p* = 0.003). The PCOS group demonstrated lower mean serum concentrations of E2 in comparison to the controls (59.89 ± 42.44 pg/ml in PCOS vs. 99.06 ± 15.34 pg/ml in controls; *p* < 0.0001). Mean serum T concentrations were 0.65 ± 0.33 ng/ml in PCOS vs. 0.33 ± 0.19 ng/ml in controls (*p* = 0.0001). PCOS patients presented higher mean serum concentrations of FBG (*p* = 0.0265). Both groups were comparable in DHEAS, TSH, fT4 and insulin mean serum concentrations (Table 2).

**Table 2:** Biochemical and hormonal characteristic of the studied and the control group

Group	PCOS (mean ± SD)	Controls (mean ± SD)	*p*
FSH (U/l)	5.88 ± 1.86	10.39 ± 0.86	0.003
LH (U/l)	12.8 ± 6.2	11.06 ± 1.12	0.73
PRL (ng/ml)	14.98 ± 9.09	8.72 ± 3.07	0.003
E2 (pg/ml)	59.89 ± 42.44	99.06 ± 15.34	<0.0001
T (ng/ml)	0.65 ± 0.33	0.33 ± 0.19	0.0001
DHEAS (µmol/l)	8.36 ± 3.95	7.53 ± 5.09	0.53
TSH (uIU/ml)	2.84 ± 1.73	2.83 ± 1.79	0.8
fT4 (ng/dl)	1.28 ± 0.16	1.3 ± 0.13	0.31
Ins (uU/ml)	8.47 ± 5.27	7.67 ± 3.71	0.14
FBG (mg/dl)	82.89 ± 17.64	75.43 ± 5.3	0.027
HOMA-IR	1.71 ± 1.36	1.4 ± 0.7	0.63

*FSH* follicle stimulating hormone, *LH* luteinizing hormone, *PRL* prolactin, *E2- 17β* estradiol, *DHEAS* dehydroepiandrosterone sulfate, *T* testosterone, *TSH* thyroid stimulating hormone, *fT4* free thyroxine, *Ins* insulin, *FBG* fasting blood glucose, *HOMA-IR* homeostatic model assessment-insulin resistance index

The mean mFG score was higher in PCOS (*p* = 0.0044). Hirsutism, with a mean mFG score equal or higher than 8 was found in 54 women with PCOS, but in only 5 women in the control group (see Table 3).

**Table 3:** Hirutism in PCOS and the control group

Group	PCOS	Controls
mFG score (mean ± SD)	18.31 ± 6.07*	6.57 ± 3.39
mFG <8 score	*n* = 6	*n* = 25
mFG ≥8 score	*n* = 54	*n* = 5

*mFG* modified Feriman-Gallwey scale

* *p* value <0.01, PCOS vs. controls

The PCOS women had significantly lower BMD values as compared to the controls (1.057 ± 0.1260 g/cm^2^, vs. 1.210 ± 0.1805 g/cm^2^, *p* < 0.0002). Women with PCOS had lower Z-score values in comparison to the control group women, appropriately −0.85 ± 1.15 vs. **−**0.13 ± 0.83.

It was evaluated whether the differences in body mass index (BMI) would have effects on the bone mass. The bone parameters between the normal weight PCOS women, overweight/obese PCOS women and control women are demonstrated in Table 4. The PCOS normal weight subgroup of patients had significantly lower BMD values than the normal weight control women (*p* = 0.0049). In the subgroup of overweight/obese PCOS women, the BMD values were not significantly different in comparison to the normal weight PCOS women and control subjects. Z-score values were comparable in all three subgroups. In the subgroup of obese PCOS patients, a significantly negative correlation between hirsutism and BMD values (*r* = −0.52, *p* = 0.04) was indicated. No significant correlations were found between bone parameters and other biochemical parameters in the subgroup of the normal weight PCOS women.

**Table 4:** BMD and Z-SCORE values in study subgroups: normal weight PCOS women, overweight/obese women, normal weight control women

Subgroup	Normal weight PCOS	Overweight/obese PCOS	Controls
Number	*n* = 41	*n* = 17	*n* = 30
BMD (g/cm^2^), (mean ± SD)	1.045 ± 0.11**	1.09 ± 0.16	1.21 ± 0.18**
Z-score, (mean ± SD)	−0.89 ± 1.03	−0.70 ± 1.46	−0.13 ± 0.83

*BMD* bone mineral density in the lumbar spine, Z-score-the number of standard deviations by which a patient value differs from the age- and gender-matched, normal reference, Polish population

** *p* value <0.01, normal weight PCOS vs. the normal weight control subgroup

**Fig. 1:**
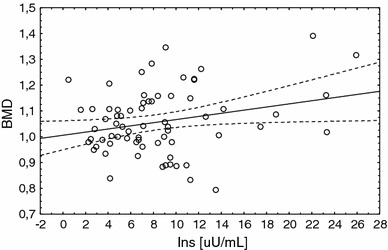
The correlation between BMD and serum insulin concentrations in PCOS patients (*r* = 0.25, *p* = 0.0347). *BMD* bone mineral density, *Ins* Insulin

In patients with PCOS, BMDs were positively correlated with insulin concentration (*r* = 0.25, *p* = 0.0347) and HOMA–IR (*r* = 0.29, *p* = 0.017; Fig. 1). No significant correlations were found between the Z-score and metabolic parameters. In the controls the Z-scores were positively correlated with insulin concentration (*r* = 0.55, *p* = 0.035) and HOMA–IR (*r* = 0.60, *p* = 0.019).

In women with PCOS, HOMA–IR significantly correlated with the BMI (*r* = 0.57, *p* < 0.001). No significant correlations were found between HOMA–IR and other metabolic parameters in the controls.

## Discussion

In our study we evaluated the serum levels of hormones such as FSH, LH, E2 and insulin. According to the literature, there is a characteristic profile of the hormones listed above in patients with PCOS [1, 13–15]. Generally, these patients are characterized by higher serum LH levels than normal subjects and similar serum FSH levels as normal subjects. Serum E2 levels in the PCOS are comparable to healthy women. Contrary serum insulin levels are higher than in the controls [1, 2, 4]. In our study, serum FSH levels in the PCOS patients were significantly lower than in the controls. This can result from the fact that the blood for the hormonal assay was taken at the late follicular phase (10th day of the menstrual cycle). The reduction in the FSH concentration in this phase is commonly associated with ovulatory disturbances among women with PCOS [6, 16]. There was no significant difference between serum LH levels in PCOS patients and the control. Similarly, it also can result from the methodological approach to the withdrawal of blood for the hormonal evaluation in the late follicular phase. On the other hand, according to some studies, about 40 % of women with PCOS have normal serum LH concentrations [15, 17]. The same problem refers to serum E2 levels, which was higher in normal subjects in comparison to the PCOS individuals. This results from the preovulatory peak of this hormone in healthy women in the late follicular phase. Surprisingly, there was no difference in serum insulin levels between PCOS patients and controls [4]. However, PCOS patients were characterized by a significantly higher HOMA index than the normal subjects.

In the present study we revealed significantly lower BMD values in the lumbar spine in a group of women with PCOS compared with healthy aged-matched controls, especially in the subgroup of normal weight women with PCOS. These results support the findings of Yüksel et al. [6] and Kirchengast et al. [16]. Most prior studies suggest that the BMD is increased in women with PCOS [7, 10, 18–21] or that total BMD does not differ significantly in PCOS subjects compared with healthy controls [18, 22]. Probably, the causes of the discrepancies we have found are the different inclusion criteria of PCOS women, as well as differences in age, BMI and menstrual irregularities among the examined women [2, 23].

Many of previous studies were performed in women with excessive body weight [21, 22, 24, 25]. In the present study, the PCOS patients were divided into subgroups according to their BMI, to assess the impact of the body mass index on the bone parameters. The results of this study found significant differences in BMD values between the subgroup of normal weight PCOS women and the control women. Between the subgroups of overweight/obese PCOS women and normal weight PCOS women and between the overweight/obese PCOS women and control women, BMD values were statistically the same. The BMI is postulated as one of the most important determinants of BMD in PCOS. The postulated mechanisms of action of the excessive body weight on bone are the insulin resistance, increase of the biomechanical forces, and increased conversion of androgens to estrogens [26, 27]. In the present study, the BMI was similar in the PCOS and control groups and ranged below 25. The results of this study do not find a significant correlation between BMD or the Z-score and BMI in the PCOS women and the controls.

It has been discussed that menstrual cycle abnormalities might also affect bone metabolism. Among females with PCOS and amenorrhea or oligomenorrhea, estrogen concentrations are lower than in healthy females and stay consistent throughout the menstrual cycle [6, 28]. It is known that in women with hypoestrogenism due to gonadal dysgenesis or hypothalamic amenorrhea the bone deterioration advances rapidly [29, 30]. Interestingly, it has been proven that PCOS women with amenorrhea have a higher bone mass compared with patients with other causes of amenorrhea [6, 18, 19]. Furthermore, disturbances of ovulation may have an influence on bone metabolism through the progesterone deficiency. Progesterone promotes bone tissue accumulation and accelerates remodeling [11]. Therefore, anovulation and/or oligovulation, which are in the diagnostic criteria of PCOS may be a negative factor for the attained bone mass and BMD.

The results of this study indicate that BMD in PCOS and the Z-score in controls were positively correlated with mean insulin concentration and HOMA–IR. However, contrary to other studies [4, 6, 22, 24] the mean insulin concentrations were comparable in both analyzed groups. In both groups insulin levels were within the normal range. Yüksel et al. [6] postulated that insulin resistance and/or hyperinsulinemia might be associated with the relative preservation of bone density in PCOS women. Also Noyan et al. [22], found that insulin resistance may play a role in BMD preservation in PCOS patients. Hyperinsulinemia/insulin resistance may stimulate osteoblast cells activity directly and indirectly via a suppression of the production of the sex hormone binding globulin (SHBG) and the insulin-like growth factor binding protein (IGFBP). Lower SHBG and IGFBP may be responsible for the increased bioavailability of sex hormones and insulin growth factor (IGF) [8, 13]. Among women with PCOS, insulin resistance is commonly caused by obesity, but insulin resistance may be present without obesity or being overweight. The results of the study show a significantly positive correlation between HOMA–IR and BMI in the overall PCOS group, but do not reveal this relationship in the overweight/obese subgroup of PCOS women. Obesity or being overweight and insulin resistance may act synergistically on bone metabolism and stimulate bone formation.

PCOS is characterized by an excessive ovarian production of androgens. This study shows statistically significant higher mean testosterone concentrations in women with PCOS. However, elevated testosterone concentrations were not significantly correlated with BMD or the Z-score. Moreover, in the subgroup of overweight/obese PCOS patients hirsutism, a clinical sign of hyperandrogenism, was negatively correlated with BMD values. This finding is in contrast to some of the previous studies’ results. Previous studies have focused on the positive role of hyperandrogenism on bone mass [10, 16, 31]. Some reports have suggested that amennorheic women with hirsutism had a higher BMD than non-hirsute amennorheic women [20, 21] Other studies indicate a positive correlation between BMD and testosterone concentration in patients with PCOS [6, 7, 22]. Androgens have been shown to act on bone metabolism directly through androgen receptors, as well as indirectly via aromatization to estrogens in the extra glandular tissues. It has been proven that dihydrotestosterone, non-aromatazable androgen increases the formation, differentiation and maturation of osteoblasts [32]. Human osteoblast-like cells are able to convert testosterone into dihydrotestosterone due to 5-α-reductase activity [10, 33]. It has been suggested that androgens may reduce interleukin-6 production, inhibit the production of prostaglandins and suppress the effect of parathyroid hormone on osteoblasts [10]. Nevertheless, many of the prior studies were performed on overweight and obese PCOS women [20, 21]. The observed higher bone density may be caused by excessive body weight. Kirchengast et al. [16], claimed that lean PCOS women had a significantly higher amount of body fat and significantly lower BMD than lean controls. Further studies, which take these differences into account, will need to be undertaken.

In conclusion, women with PCOS have a significantly lower BMD of the lumbar spine compared to controls. There is no significant correlation between testosterone and BMD in young women with PCOS, but a significant negative correlation between hirsutism and BMD in the subgroup of overweight PCOS women has been proven. Bone mass parameters in both the examined groups are positively correlated with mean insulin concentration and HOMA–IR. Insulin appears to be one of the most important positive bone growth stimulators. The molecular mechanism of insulin influence on bone formation needs to be further studied. The BMI is another important determinant of BMD in PCOS women.
